# Prospective Evaluation of the Safety and Compression Performance of Novel Compression Denim Jeans in Healthy Volunteers and Patients With Lymphedema

**DOI:** 10.7759/cureus.80971

**Published:** 2025-03-21

**Authors:** Daiki Ousaka, Kiyoshi Yamada, Noriko Sakano, Satoe Kirino, Kazumasa Miyake, Takumi Takahashi, Akihiro Matsuoka, Shintaro Yamada, Akira Shinaoka, Susumu Oozawa

**Affiliations:** 1 Department of Pharmacology, Okayama University Graduate School of Medicine, Dentistry and Pharmaceutical Sciences, Okayama, JPN; 2 Departments of Plastic and Reconstructive Surgery, Okayama University Graduate School of Medicine, Dentistry and Pharmaceutical Sciences, Okayama, JPN; 3 Department of Cardiovascular Surgery, Okayama University Graduate School of Medicine, Dentistry and Pharmaceutical Sciences, Okayama, JPN; 4 Department of Obstetrics and Gynecology, Okayama University Graduate School of Medicine, Dentistry and Pharmaceutical Sciences, Okayama, JPN; 5 Department of Rehabilitation, Lymphedema Treatment Center, Kousei Hospital, Okayama, JPN; 6 Division of Business Management, Matsuoka Corporation, Hiroshima, JPN; 7 Division of Production Engineering, Matsuoka Corporation, Hiroshima, JPN; 8 Division of Sales, Kaihara Corporation, Hiroshima, JPN; 9 Department of Lymphatics and Edematology, Okayama University Graduate School of Medicine, Dentistry and Pharmaceutical Sciences, Okayama, JPN; 10 Department of Clinical Safety, Okayama University Hospital, Okayama, JPN

**Keywords:** compression garments, denim jeans, long-term management, lower-extremity lymphedema, quality of life

## Abstract

Objectives: The treatment of lower-extremity lymphedema, whether congenital or acquired, remains challenging. Long-term management aimed at reducing complications and maximizing quality of life is essential. Compression stockings are crucial in this management; however, their application is limited by patient experience (ease of wear, texture, breathability, and appearance). This highlights the need to evaluate alternative compression garments that maintain therapeutic efficacy while improving patient adherence.

Methods: We developed a novel compression denim product (Flow plus Jeans®) using advanced sewing technology. Its baseline performance (compression ability) was evaluated by measuring pressure gradients at three points (ankle, calf, and thigh) using a mannequin-based compression testing system and compared with those of existing stockings. Thereafter, a safety assessment was conducted on healthy volunteers to evaluate potential adverse effects, including changes in lower limb circumference, signs of deep vein thrombosis (DVT) via ultrasound, and skin complications. A clinical trial in patients with lymphedema was then performed to compare its efficacy with that of conventional compression stockings.

Results: Baseline performance testing with a mannequin revealed that Flow plus Jeans demonstrated compression levels and pressure gradients at three calf points comparable to those of standard compression stockings. A safety study involving nine healthy volunteers confirmed that Flow plus Jeans caused no significant changes in lower-limb circumferences after three days of wear, with no cases of DVT or skin complications. In a subsequent clinical trial involving nine female patients with lymphedema, the jeans showed non-inferiority to existing stockings concerning lower-limb circumference measurements at six points (pre-use vs. six months post-use), with patient-reported experiences assessed via questionnaires. Notably, patients reported enhanced satisfaction regarding the jeans' fashionability, which could serve as an incentive for long-term adherence.

Conclusion: Our findings suggest that Flow plus Jeans represent a promising novel option for the long-term management of lymphedema, offering an alternative that balances medical efficiency with improved patient satisfaction and demonstrates safety in healthy individuals.

## Introduction

Lymphedema can be classified into primary (congenital or genetic causes) and secondary (resulting from infections, trauma, or surgical and radiation treatments) forms, and it usually causes significantly impaired quality of life [1]. Notably, lower-extremity lymphedema constitutes approximately 74% of cases, and its treatment and management strategies are broadly divided into (i) surgical interventions such as lymphovenous anastomosis, lymph node transplantation, and liposuction, and (ii) non-surgical therapies including compression therapy, exercise therapy, skincare, and anti-inflammatory medications. Secondary lower-extremity lymphedema disproportionately affects women, with a male-to-female ratio of 1:9 [2-5]. Regardless of the treatment modality, achieving a complete cure remains challenging, making long-term management and adherence critical. Consequently, maintaining patient motivation and ensuring robust support systems are essential.

In our clinical practice, we have primarily managed patients with lymphedema through long-term compression therapy using medical compression stockings. However, we received consistent complaints, particularly from female patients, regarding the shortcomings of these stockings, such as their appearance, clothing restrictions, discomfort, and complications with donning and doffing. To address these challenges and enhance long-term treatment adherence, we developed a novel compression denim product, Flow plus Jeans®, which is a registered trademark of Stork Visit Co., Ltd. (Okayama, Japan) [6]. This product features compression force and gradient comparable to that of medical compression stockings, is made of denim material, and has a stylish appearance resembling skinny jeans.

To assess the clinical utility of Flow Plus Jeans, the study evaluated its compression performance and compared its usability with conventional medical stockings. In addition to objective measurements, patient-reported surveys were conducted before and after the trial to assess factors such as fashionability and wearing comfort. This study aims to explore whether Flow Plus Jeans can provide a viable alternative to traditional compression therapy by addressing both therapeutic efficacy and patient adherence challenges.

## Materials and methods

The study was conducted at Okayama University Hospital, Okayama City, Japan, from 2018 to 2024.

Development of Flow plus Jeans

The jeans were developed to address the shortcomings of medical compression stockings commonly used for managing lower-extremity lymphedema, particularly regarding fashionability and comfort. Using a specialized sewing technique, a graduated compression function was integrated into denim jeans. The manufacturing method and technical innovations of Flow plus Jeans are detailed in the corresponding patent application [6]. To ensure an optimal fit and effective compression, each pair of Flow plus Jeans was custom-made for individual participants based on their lower limb circumference measurements.

Basic performance testing: three-point measurement on a mannequin

To evaluate the basic performance of Flow plus Jeans, compression force was measured at three points on the lower leg (ankle, distal to the calf, and posterior calf) using an air-pack-based garment pressure measurement device (AMI Techno, Tokyo, Japan) and compared with JOBST compression stockings (Essity AB, Stockholm, Sweden), a widely used conventional medical compression stocking.

Clinical trial for healthy volunteers

The clinical trial was registered in the University Hospital Medical Information Network Clinical Trials Registry (UMIN-CTR) as an interventional study (registration number: UMIN000028657) and was approved by the Okayama University Hospital Ethics Committee (approval number: 1709-005). It was conducted between April 1, 2018, and March 31, 2019. 

Participants

Nine healthy participants (three males and six females) with an average age of 38 years were included on meeting the following inclusion criteria: healthy adults (i) with no history of vascular, lymphatic, or neuromuscular disorders affecting the lower limbs, (ii) aged 20-60 years, (iii) willing to participate in the study and adhere to the protocol, (iv) with no prior history of deep vein thrombosis (DVT) or circulatory disorders, and (v) able to wear Flow plus Jeans continuously during daytime hours for three consecutive days. Participants were excluded if they (i) had chronic diseases affecting circulation (e.g., cardiovascular disease, peripheral artery disease, or chronic venous insufficiency), (ii) had a recent lower-limb injury or surgery within 6six months prior to enrollment, (iii) had known allergies or skin sensitivities to the materials used in Flow plus Jeans, (iv) were currently using compression therapy for any medical condition, (v) were pregnant or planning pregnancy during the study period, or (vi) were unable or unwilling to comply with the study protocol. 

Measurements and Assessments

All participants underwent lower-limb circumference measurements at three points (ankle, calf, and thigh) to assess potential changes associated with Flow plus Jeans use. Measurements were taken in the morning before wearing the jeans and in the evening after three consecutive days of daytime use. The measurement sites were selected based on anatomical landmarks to ensure consistency, with the ankle measured at its narrowest part, the calf at its thickest part, and the thigh at its thickest part. While these locations allowed for relatively uniform measurements across participants, no physical markings were made to ensure exact placement, which has been acknowledged as a limitation.

Additionally, complication assessments were conducted at the end of the study, including ultrasound evaluation of the lower-limb veins to screen for DVT and visual inspection of the skin for any irritation, rashes, or other dermatological issues related to wearing Flow plus Jeans.

Clinical trial for patients with lymphoedema

The clinical trial was approved by the Okayama University Hospital Ethics Committee (approval number: 2106-045) and was conducted between July 9, 2021, and August 30, 2024. During the study period, all eligible patients who visited the vascular surgery outpatient clinic and met the inclusion criteria were invited to participate. Those who provided informed consent were enrolled. No prior sample size calculation was performed.

Participants

Nine female participants with an average age of 56 years were included on meeting the following inclusion criteria: (i) diagnosis of lower-limb lymphedema following surgery for uterine cancer, confirmed by a certified medical professional, (ii) stable clinical condition without significant progression or acute complications of lymphedema, (iii) aged 30-70 years, and (iv) willingness to participate in the study and adherence to the protocol. Participants were excluded if they met any of the following criteria: (i) presence of severe comorbidities such as cardiovascular, pulmonary, or renal diseases, (ii) active skin conditions such as infections, ulcers, or dermatitis in the affected limb, (iii) known allergies to the materials used in Flow plus Jeans, (iv) recent surgery or injuries to the lower limbs within six months prior to enrollment, or (v) inability or unwillingness to comply with the study protocol.

Measurements and Assessments

All participants underwent an initial evaluation of lymphedema management using conventional medical compression stockings. Lower-limb circumferences were measured at six points from the abdomen to the lower limbs by hospital staff during morning outpatient visits, ensuring consistency in assessment. Measurement sites were clearly defined, allowing for consistent placement before and after wearing the compression stockings. In cases of bilateral lymphedema, the limb with the more severe swelling was selected for measurement to ensure the assessment captured the most clinically relevant side. While this approach helped maintain measurement accuracy, no physical markings were made to ensure exact placement, which has been acknowledged as a limitation.

Questionnaire-Based Analysis of User Satisfaction and Experience

Subsequently, the same participants were evaluated for the efficiency of Flow plus Jeans. To evaluate the user satisfaction and experience of wearing Flow plus Jeans compared with that of conventional medical compression stockings, a questionnaire survey (see Appendices) was conducted with seven assessment items: fit and comfort, compression sensation, fashionability, ease of wear, skin irritation, price range, and overall satisfaction. Participants completed the questionnaire at two time points: (i) immediately after wearing Flow plus Jeans for the first time, and (ii) after six months of continuous use. For each item, a scoring system based on a relative scale was employed. Conventional medical compression stockings were assigned a baseline score of 5 points, and participants rated Flow plus Jeans on a 0-to-10-point scale, where 0 points indicated "much worse than conventional stockings," 5 points indicated "equivalent to conventional stockings," and 10 points indicated "much better than conventional stockings."

Scores for each item were collected and analyzed to evaluate user perceptions of the new product relative to the standard treatment. The mean scores for each category were calculated at both time points to assess changes in satisfaction and experience over time.

Statistical analysis

Statistical analyses were conducted using GraphPad Prism Software version 9.5.1 (Dotmatics, Boston, Massachusetts). For the basic performance test, compression forces within each group (stockings and Flow plus Jeans) at different measurement points (ankle vs. distal to the calf, or posterior calf) were compared using a paired t-test. Similarly, comparisons of lower-limb circumferences before and after six months of use in the clinical trial were performed using a paired t-test. User satisfaction and experience surveys were analyzed with an unpaired t-test to compare the two groups (stockings vs. Flow plus Jeans) at pre-use and six-month follow-up. In the healthy volunteer group, lower-limb circumferences before and after three days of use were also compared using a paired t-test. A value of p < 0.05 was considered statistically significant.

## Results

Development of Flow plus Jeans

**Figure 1:**
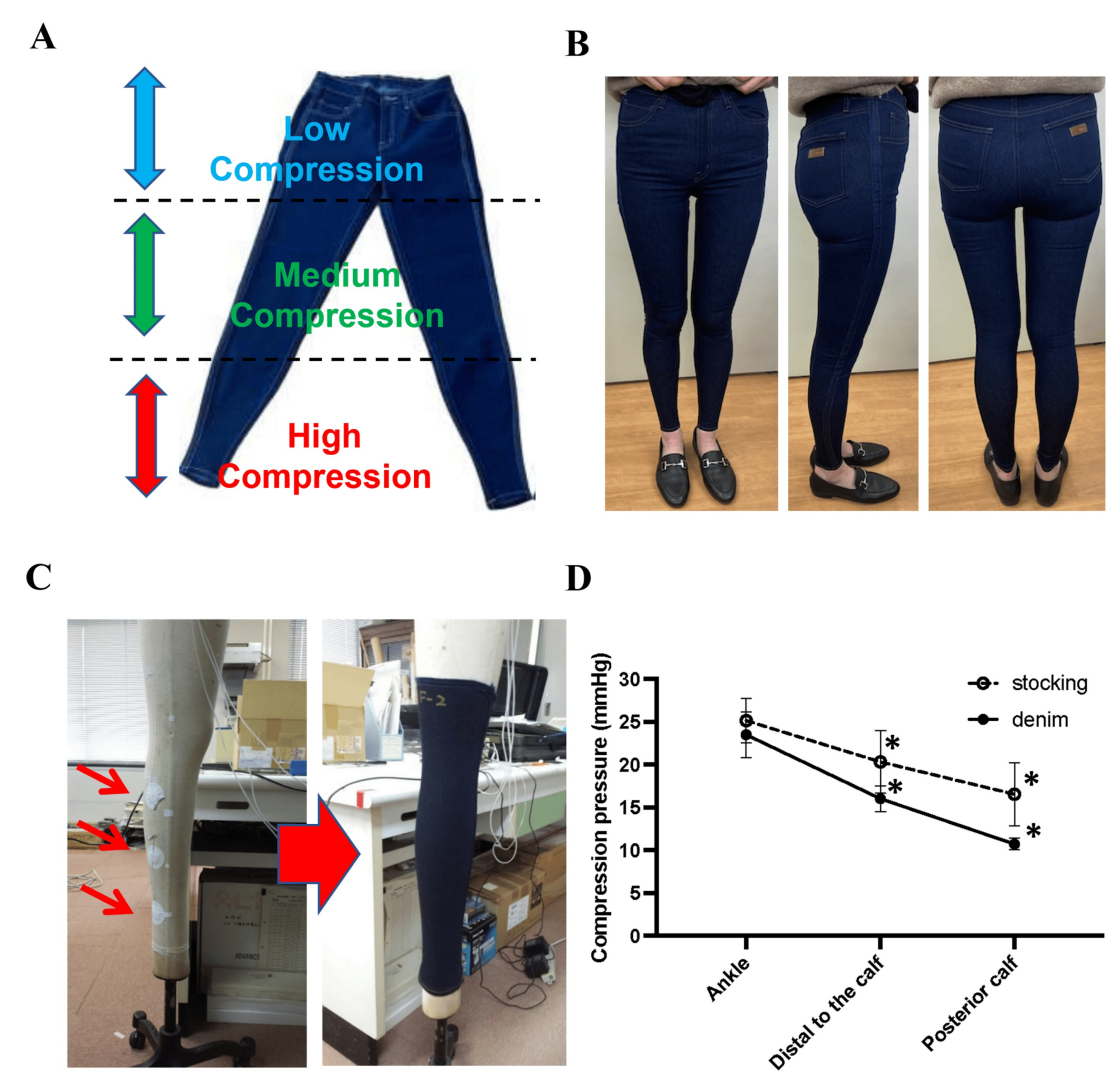
Development of denim-style compression jeans using novel sewing technology (A) Overview of Flow plus Jeans® developed using proprietary sewing technology (patent granted). The jeans appear identical to standard stretch jeans but are tailored to provide a graduated compression effect, with pressure gradually decreasing from the ankle to the abdomen. (B) Photographs demonstrating the appearance and fit of Flow plus Jeans® when worn. The jeans resemble standard skinny jeans while incorporating a graduated compression function designed for lymphedema management. These images provide a visual reference for the product’s integration into everyday wear. (C) Results of compression gradient testing using a mannequin. Pressure sensors placed at three points on the lower leg measured compression forces for Flow plus Jeans® and conventional medical compression stockings. (D) Both products exhibit a gradual decrease in compression pressure from the ankle toward the proximal side. Statistical analysis using a t-test showed no significant differences between the two groups at any measurement point. p < 0.01: comparison to the ankle within each group (n = 3). This figure represents the within-group comparisons of ankle vs. distal to the calf and ankle vs. posterior calf for each group separately. The asterisk (*) indicates significant differences in these within-group comparisons (p < 0.01). Error bars indicate standard deviation (SD).

The Flow plus Jeans is shown in Figures 1A, 1B. The denim is tailored to provide a graduated compression effect, with pressure gradually decreasing from the lower leg to the abdomen. The jeans maintain the appearance of standard skinny jeans, achieving high fashionability.

Basic performance testing: three-point measurement on a mannequin

Compression measurements using a mannequin confirmed the non-inferiority of Flow plus Jeans to conventional medical compression stockings concerning therapeutic compression force and gradual pressure change (Figure 1C, 1D). The measured interface pressures were as follows: Stockings: ankle: 25.2 ± 4.5 mmHg, distal to the calf: 20.3 ± 6.3 mmHg, posterior calf: 16.6 ± 6.4 mmHg, and Flow plus Jeans: ankle: 23.5 ± 4.6 mmHg, distal to the calf: 16.0 ± 2.7 mmHg, posterior calf: 10.7 ± 1.2 mmHg. A statistical comparison between the two groups using an unpaired t-test showed no significant difference in compression pressure. These measurements were conducted on three different product samples (n = 3), with each sample tested once per measurement site, ensuring that variations between products were accounted for rather than repeated testing of a single product.

Clinical trial for healthy individuals

**Figure 2:**
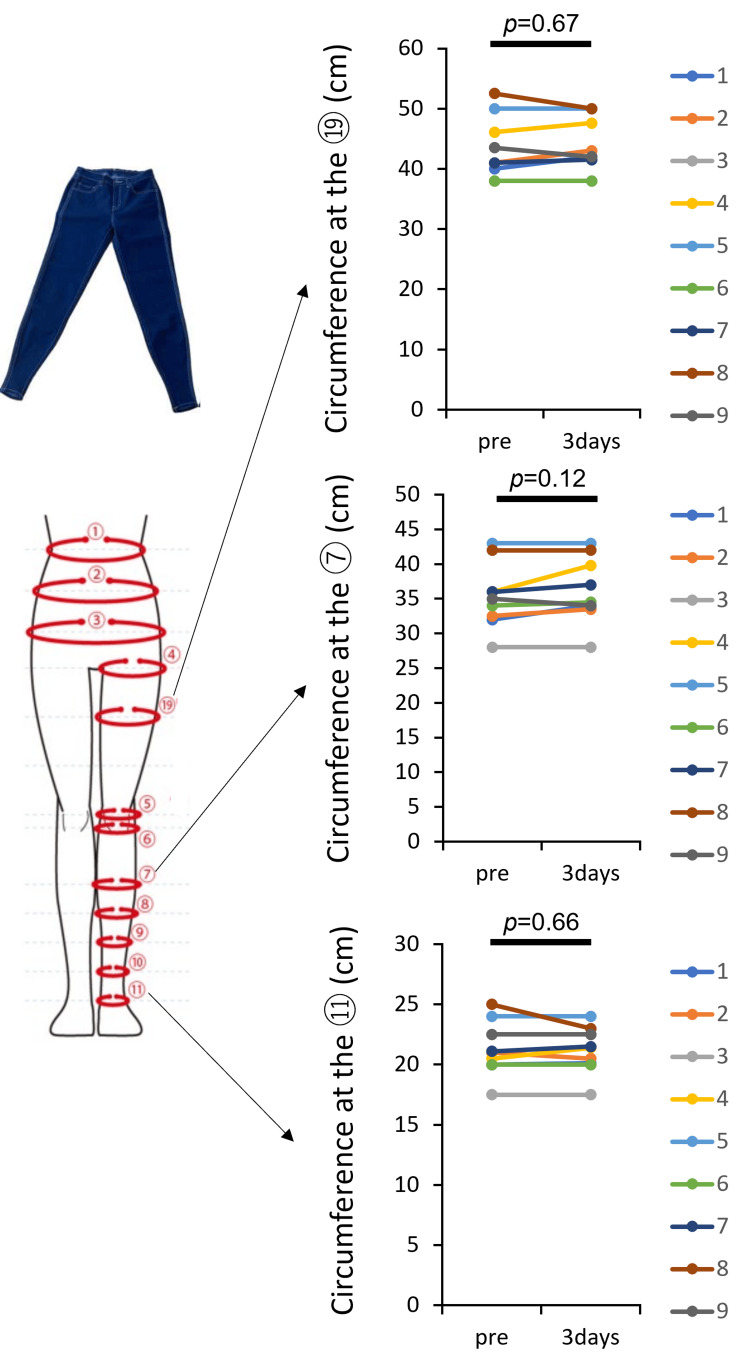
Safety evaluation of Flow plus Jeans® in healthy participants A study was conducted in nine healthy individuals to primarily assess the safety of Flow plus Jeans®. The lower image presents changes in lower-limb circumferences at the ankle (⑪), calf (⑦), and thigh (⑲) before wearing and after three days of use. No significant changes in limb circumference were observed at any measurement site. Statistical analysis was performed using a paired t-test, and no significant differences were detected between pre-use and post-use measurements (p > 0.05).

Nine healthy participants (three male and six female participants, mean age 38 years) successfully completed the study protocol without any withdrawals or deviations. Statistical analysis showed no significant changes in lower-limb circumferences across all measurement points, indicating that wearing Flow plus Jeans did not cause lower-limb swelling or any adverse circulatory effects in healthy individuals (Figure 2).

Additionally, to evaluate potential complications, ultrasound examinations of the lower-limb veins were conducted at the end of the study, confirming the absence of DVT in all participants. Furthermore, no skin-related adverse events such as irritation, rashes, or discomfort were reported, as confirmed by visual skin assessments.

Overall, these findings suggest that Flow plus Jeans did not induce any adverse effects in healthy individuals, including circulatory impairment or skin complications, during the three-day wear period. These results support the safety profile of Flow plus Jeans for extended use in a general population.

Clinical trial for patients with lymphoedema

**Figure 3:**
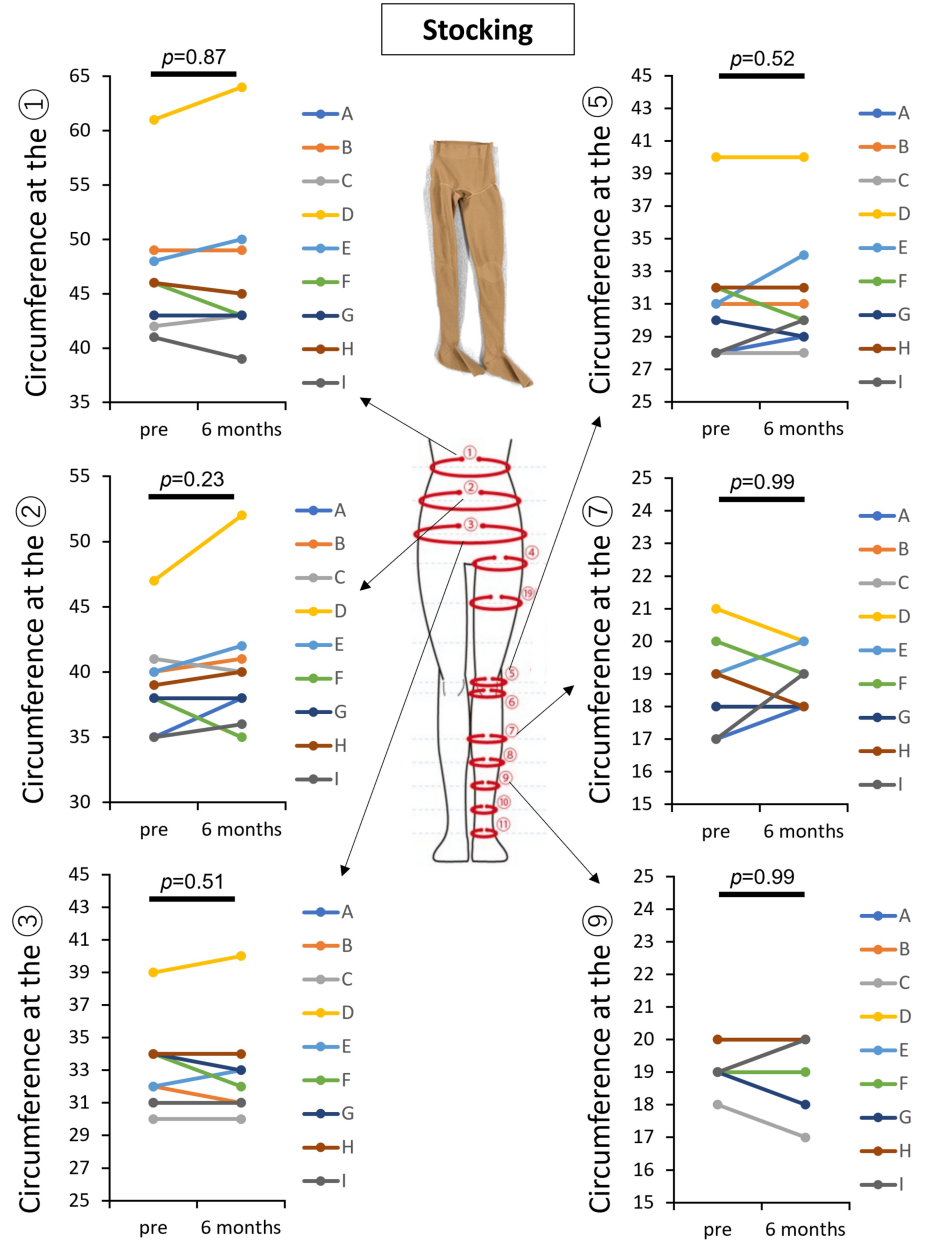
Assessment of conventional medical compression stockings in study participants with lymphedema Changes in circumferences from the abdomen to the lower limbs before and after six months of compression therapy using conventional medical stockings in nine patients with lymphedema (A–I). Measurements were taken at six points (①, ②, ③, ⑤, ⑦, and ⑨). Statistical analysis using a paired t-test showed no significant increases in circumference between pre-treatment and six-month follow-ups (p > 0.05), indicating that compression therapy effectively managed lymphedema without worsening.

**Figure 4:**
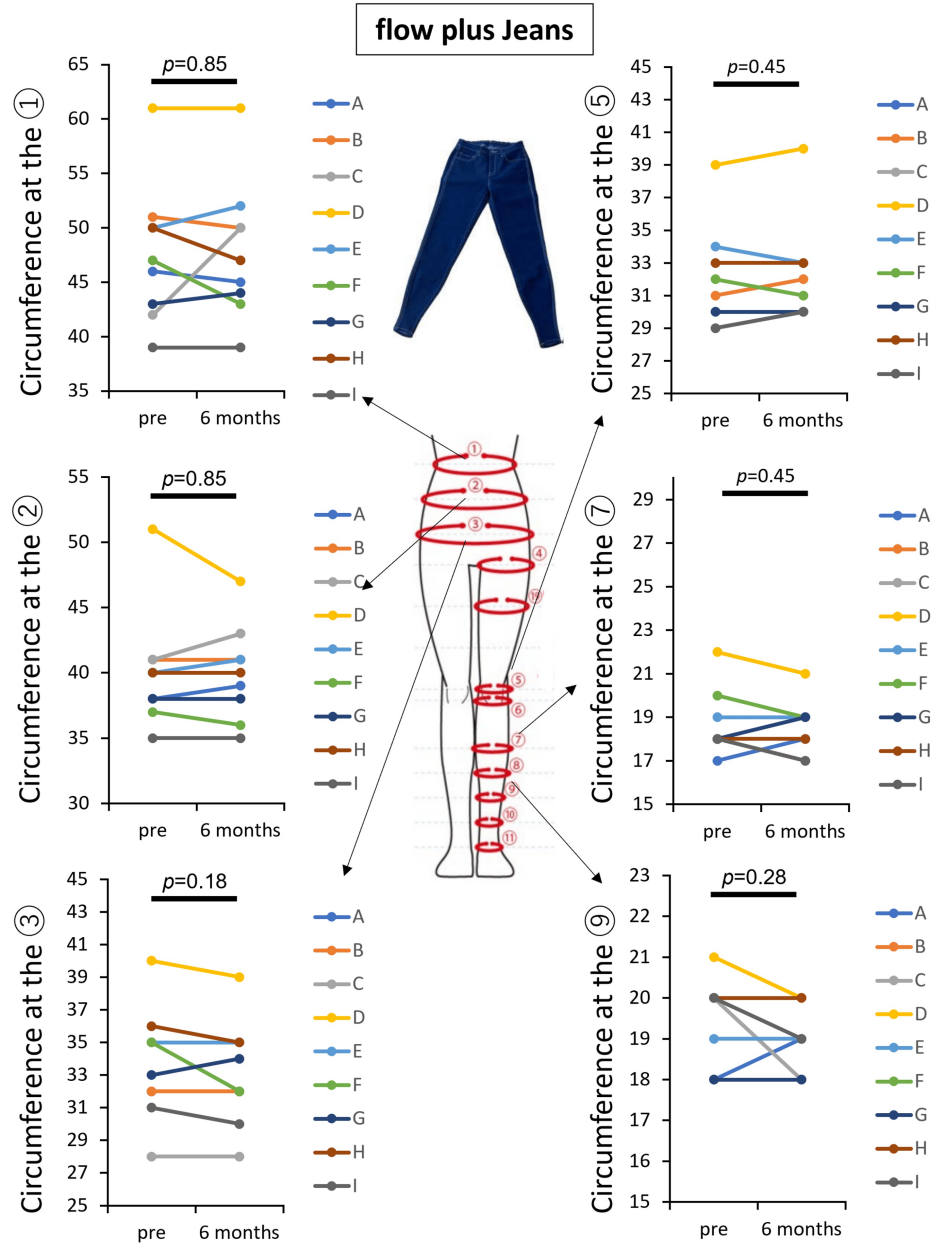
Assessment of Flow plus Jeans® in study participants with lymphedema Changes in circumferences from the abdomen to the lower limbs before and after six months of compression therapy using Flow plus Jeans® in nine lymphedema patients (A–I) are shown. Measurements were taken at six points (①, ②, ③, ⑤, ⑦, and ⑨). Statistical analysis using a paired t-test showed no significant increases in circumference between pre-treatment and six-month follow-ups (p > 0.05), indicating that compression therapy effectively managed lymphedema without worsening.

First, the efficiency of conventional stockings in managing lower-extremity lymphedema was evaluated in nine participants (Table 1). Measurements of lower-limb circumferences at six points (Figure 3) before and six months after use revealed no significant increase in circumferences at any site, demonstrating the effectiveness of compression therapy. Subsequently, the same participants were evaluated for the therapeutic effectiveness of Flow plus Jeans. Similar to conventional stockings, there was no significant increase in lower-limb circumferences at six points (Figure 4) after six months of use, confirming the effectiveness of compression therapy. These results indicate the non-inferiority of Flow plus Jeans compared to conventional stockings in managing lower-extremity lymphedema.

**Table 1:** Baseline characteristics of study participants with lymphoedema

Patient	Age (years)	Sex	Weight (kg)	Diagnosis	Affected limb	Medical History
A	56	Female	44	Lymphedema	Both	Hysterectomy for cervical cancer
B	39	Female	48	Lymphedema	Right	Hysterectomy for cervical cancer
C	75	Female	46	Lymphedema	Both	Hysterectomy for cervical cancer
D	65	Female	74	Lymphedema	Both	Hysterectomy for cervical cancer
E	46	Female	58	Lymphedema	Both	Hysterectomy for cervical cancer
F	62	Female	50	Lymphedema	Both	Hysterectomy for endometrial cancer
G	38	Female	51	Lymphedema	Both	Hysterectomy for cervical cancer
H	71	Female	52	Lymphedema	Both	Hysterectomy for endometrial cancer
I	55	F	41	Lymphedema	Left	Laparoscopic surgery for ectopic pregnancy

User Satisfaction Survey

**Figure 5:**
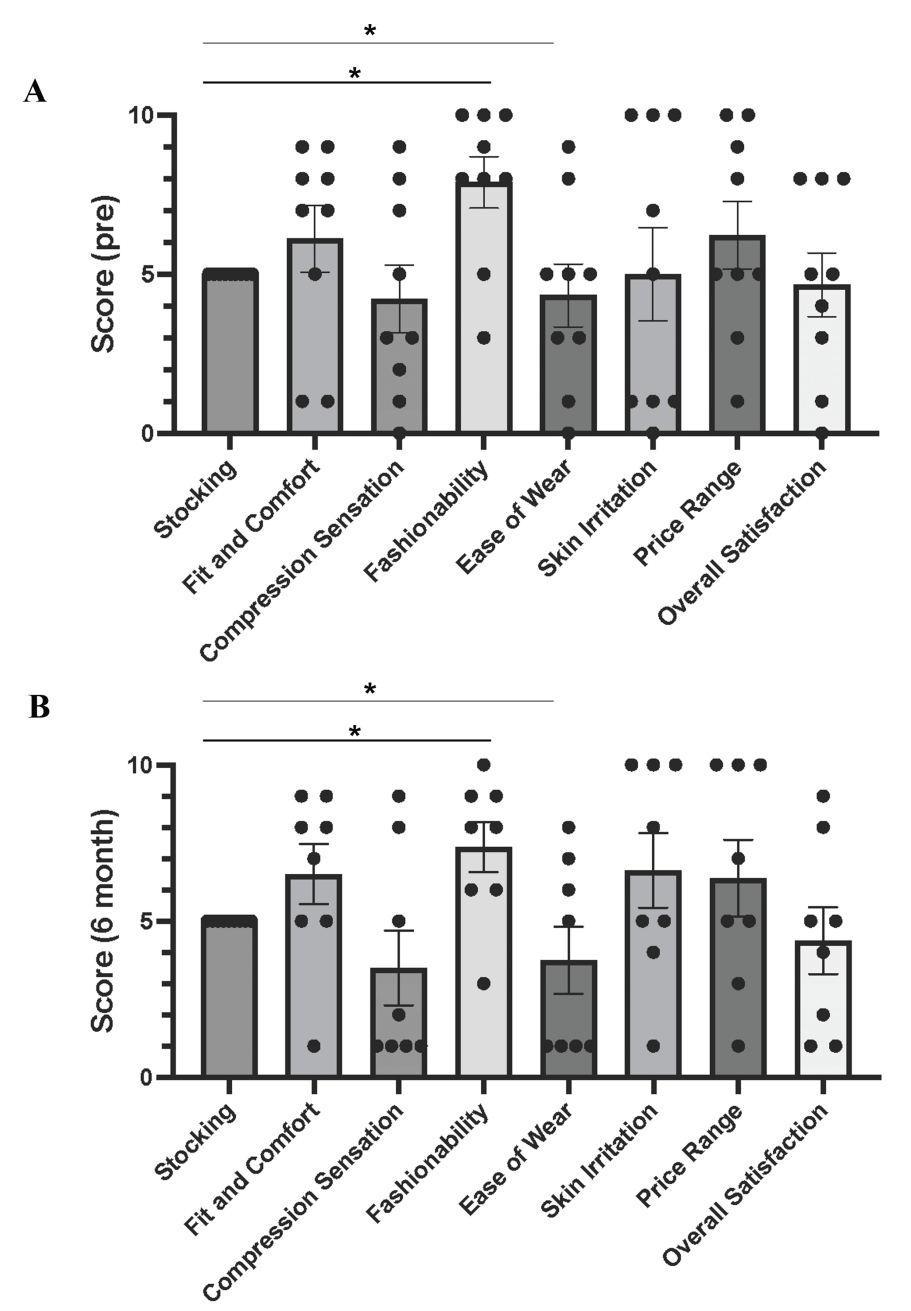
User experience survey comparing Flow plus Jeans® and conventional medical compression stockings (A, B) Results of questionnaires assessing user experience with conventional medical stockings and Flow plus Jeans® immediately after use (pre) and after six months (pre: n = 9, 6 months: n = 8). Among the seven evaluated parameters, statistical analysis using an unpaired t-test showed that Flow plus Jeans® had significantly higher satisfaction in terms of fashionability than stockings at both pre-treatment and six months (*p < 0.05). However, Flow plus Jeans® was significantly inferior to stockings in terms of ease of wear at both pre-treatment and six-month time points (*p < 0.05).

The user satisfaction and experience survey for Flow plus Jeans conducted before use and six months after starting to use indicated that Flow plus Jeans achieved higher satisfaction regarding fashionability than did conventional stockings at both time points (Figure 5A, 5B). However, the ease of wear was inferior to that of conventional stockings at pre-use and six-month follow-ups (Figure 5A, 5B). Other parameters, including fit and comfort, compression sensation, skin irritation, price range, and overall satisfaction, showed no significant differences between the two groups.

## Discussion

Several studies have confirmed that compression therapy combined with appropriate exercise is an effective management strategy for lymphedema [7-10]. Compression therapy helps reduce fluid accumulation, maintain limb volume, and prevent disease progression. However, despite its effectiveness, two primary challenges hinder its widespread and long-term use: adherence to long-term therapy and difficulty in selecting appropriate compression levels [11,12]. 

Adherence to compression therapy is often suboptimal owing to multiple factors, which can be categorized into the following five key domains. First, regarding physical factors, many patients report discomfort due to heat retention, itchiness, excessive tightness, and difficulty in donning and doffing compression garments [13]. Improper application may lead to uneven pressure distribution, potentially worsening symptoms or causing skin-related complications [14]. Second, regarding psychological factors, the daily requirement for compression garment use can lead to treatment fatigue, decreased motivation, and frustration, particularly if the perceived benefit is not immediate or obvious [15]. Third, regarding social factors, economic burden is a well-documented barrier to compression therapy, because medical-grade compression garments can be expensive and may not be fully covered by insurance [16]. Furthermore, workplace constraints can limit patients' ability to wear these garments throughout the day [17]. Additionally, in rural areas where access to specialized lymphedema care is often limited, adherence to compression therapy may be further hindered by the need for long-distance travel to medical facilities. Studies suggest that non-traditional compression garments, which integrate compression therapy into everyday clothing, may improve adherence in these populations by offering a more practical and sustainable solution [18]. Fourth, regarding cultural factors, many compression stockings have aesthetic limitations, making them conspicuous and difficult to integrate into daily fashion. This is particularly relevant for younger patients and individuals who prioritize personal appearance [19]. Fifth, regarding healthcare-related factors, a lack of structured follow-up systems in many healthcare settings can lead to inconsistent patient compliance and the improper use of compression therapy [20].

To address these barriers, a multifaceted approach is necessary, integrating technological advancements, patient education, psychological support, and financial assistance. Regarding material innovation for comfort and aesthetics, the comfort and wearability of compression garments play a crucial role in patient adherence to therapy. Developing materials that are more breathable, flexible, and lightweight can help reduce discomfort, making long-term use more feasible. Enhanced fabric technology that improves moisture management and elasticity may also contribute to better patient satisfaction. Additionally, integrating compression therapy into everyday clothing rather than maintaining a purely medical appearance could encourage greater acceptance and consistent use, particularly among individuals who prioritize both functionality and aesthetics in their daily attire.

We sought to enhance adherence by developing Flow plus Jeans, a novel denim-based compression garment that was designed to provide both effective compression therapy and improved fashionability. The findings of the current study demonstrated that Flow plus Jeans, developed using proprietary sewing technology, achieved compression levels and graduated pressure comparable to conventional medical stockings, ensuring its therapeutic efficacy. Moreover, the jeans' appearance, identical to that of standard skinny jeans, led to higher satisfaction in terms of fashionability, suggesting a potential improvement in adherence among patients who are hesitant to use traditional compression stockings. Importantly, the psychological benefit of wearing a more aesthetically acceptable garment should not be underestimated, because self-image and confidence can significantly impact treatment compliance [21].

Beyond adherence, Flow plus Jeans may also offer additional therapeutic advantages. First, its compression gradient was found to be comparable to that of medical stockings, suggesting that it may effectively contribute to lower-limb volume maintenance and edema prevention. While this study did not assess physiological markers such as microcirculation or venous return, future investigations could explore whether the fabric composition of denim-based compression garments provides additional benefits. Second, Flow plus Jeans integrates compression therapy into daily clothing, allowing for discreet and sustained compression throughout the day. Unlike conventional stockings, which patients may remove due to discomfort or aesthetic concerns, jeans-based compression may encourage more consistent wear, potentially enhancing long-term therapeutic outcomes.

However, one notable limitation identified was the ease of wear, particularly at the ankle region. Some participants reported difficulty in donning and doffing the jeans, suggesting that further modifications in material flexibility or structural design may be necessary to optimize user experience. Future iterations of Flow plus Jeans should consider incorporating more stretchable fabric blends or adaptive fastenings to improve the ease of use without compromising compression performance.

Several recent innovations in compression therapy have aimed to improve comfort and breathability. For example, Cool Lala® (Batel Plus Corporation, Tokyo, Japan) has introduced compression stockings with enhanced air permeability, addressing one of the major physical discomforts associated with conventional garments. However, these products still maintain a traditional stocking-like appearance, which remains a limitation in terms of patient adherence. To our knowledge, Flow plus Jeans is the first denim-based compression garment designed specifically for lymphedema management. Integrating compression therapy into everyday clothing represents a novel approach to improving both adherence and quality of life for patients. This innovation has the potential to reshape how compression therapy is perceived and utilized, particularly among younger and fashion-conscious patients who may otherwise be resistant to conventional treatments.

Limitations of the study

This study has some limitations. First, the sample size was relatively small (n = 9), which may limit the generalizability of the findings. Future studies with a larger cohort are necessary to confirm the effectiveness and user experience of Flow plus Jeans in a broader population. Future studies will also incorporate statistically determined sample sizes to validate the efficacy and long-term benefits in a larger cohort.

Second, the study duration was limited to six months; the long-term adherence and durability of the product require further investigation. Third, while subjective user satisfaction was assessed through questionnaires, objective measures of comfort and compliance, such as wear time tracking and real-world activity monitoring, were not included. Fourth, although circumference measurements were taken at standardized anatomical landmarks, no physical markings were made to ensure exact placement, which may have introduced variability in the measurements. Similarly, in the clinical trial with conventional compression stockings, all circumference measurements were conducted by hospital staff during morning outpatient visits, but no physical markings were applied to ensure precise consistency between pre- and post-treatment measurements.

Fifth, while both Flow plus Jeans and conventional compression stockings prevented an increase in lower-limb circumference, neither resulted in a significant reduction. Although this suggests that both products helped maintain limb volume, a more precise efficacy assessment would require a control group without compression therapy. However, establishing an untreated lymphedema patient group is ethically and practically challenging.

Sixth, foot swelling was not specifically assessed in this study as Flow plus Jeans does not cover the foot. While no visible swelling or patient complaints were observed during clinical follow-ups, future studies should include foot circumference measurements to ensure a comprehensive evaluation of compression effects.

Seventh, significant variability was observed in patient satisfaction scores, suggesting that Flow plus Jeans may be more suitable for certain subpopulations. Further studies are needed to establish appropriate patient selection criteria. Additionally, individual compression pressures were not measured, as the jeans' design allowed for sewing adjustments based on the lower-limb circumference to provide a degree of pressure control. However, the lack of direct compression pressure measurements may have contributed to the observed variation in satisfaction scores. Future studies should incorporate individual compression pressure assessments to clarify their impact on patient experience and treatment outcomes.

Lastly, the study focused exclusively on female participants in the trial for participants with lymphedema, and the usability and efficacy of Flow plus Jeans in male patients remain to be explored.

## Conclusions

We developed a novel compression therapy device, Flow plus Jeans, in the form of denim jeans for managing lower-extremity lymphedema. The study demonstrated its functional non-inferiority to conventional medical compression stockings. While further refinements are needed, Flow plus Jeans offers a highly fashionable alternative and holds promise as a new option for lymphedema management.

## Figures and Tables

**Figure 6:**
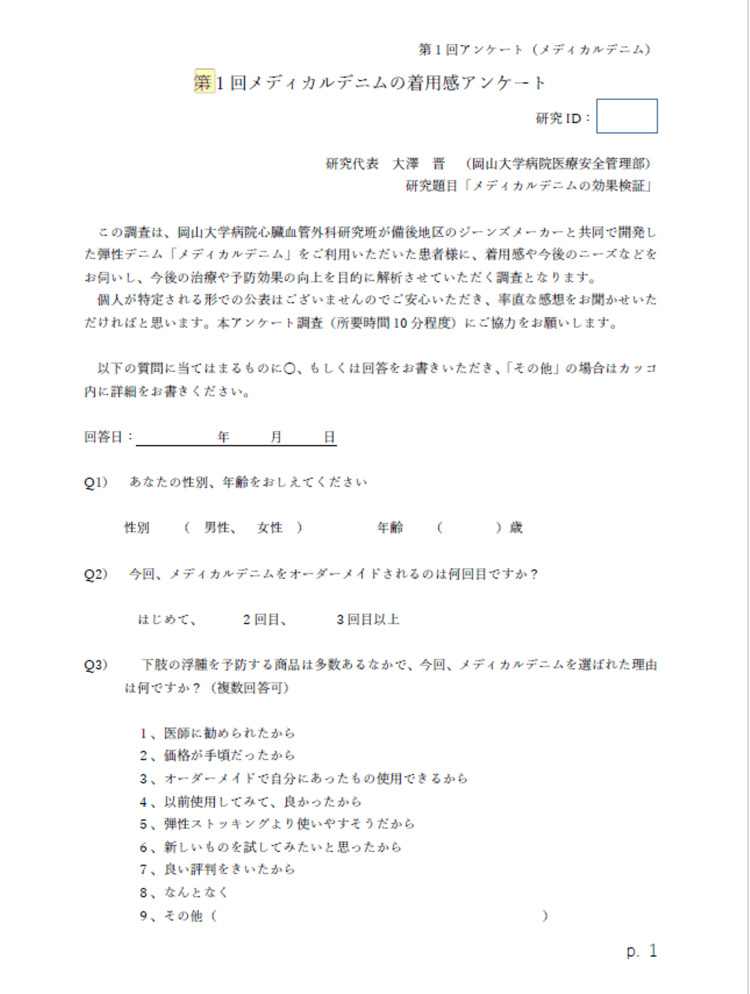
Japanese Questionnaire Page 1

**Figure 7:**
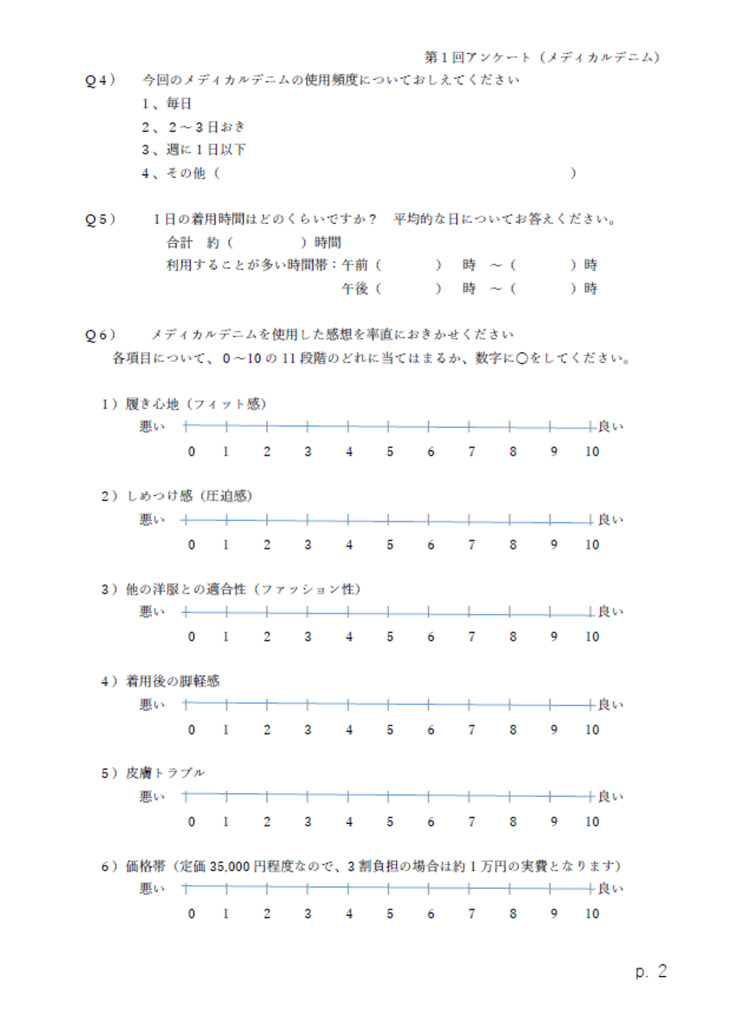
Japanese Questionnaire Page 2

**Figure 8:**
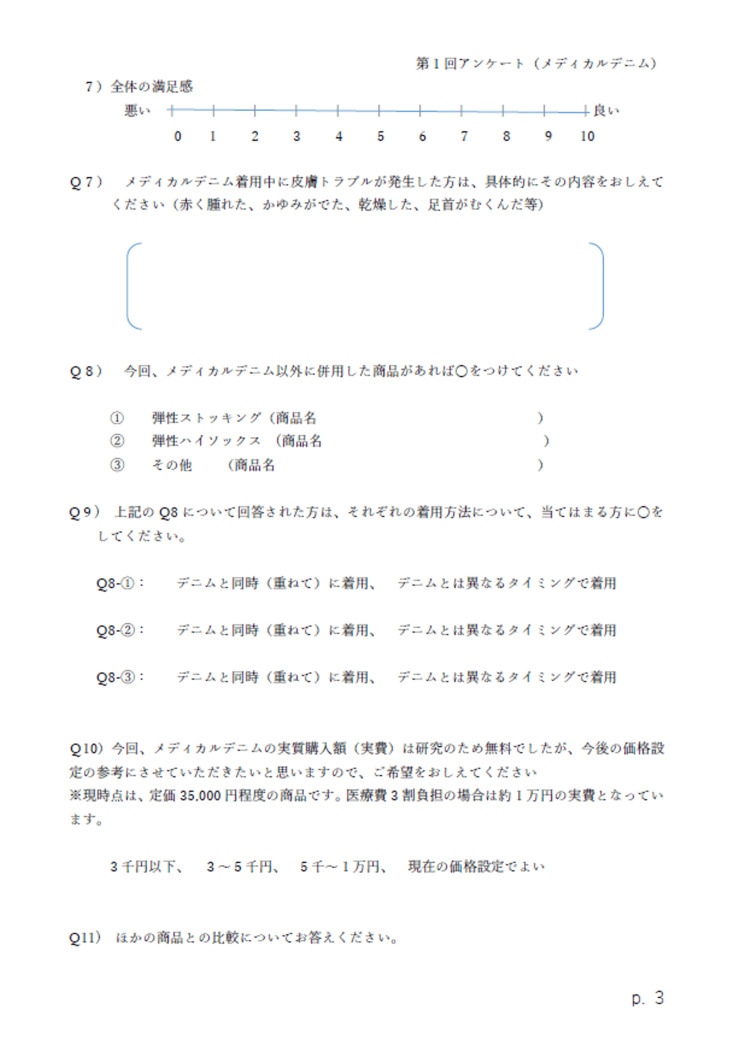
Japanese Questionnaire Page 3

**Figure 9:**
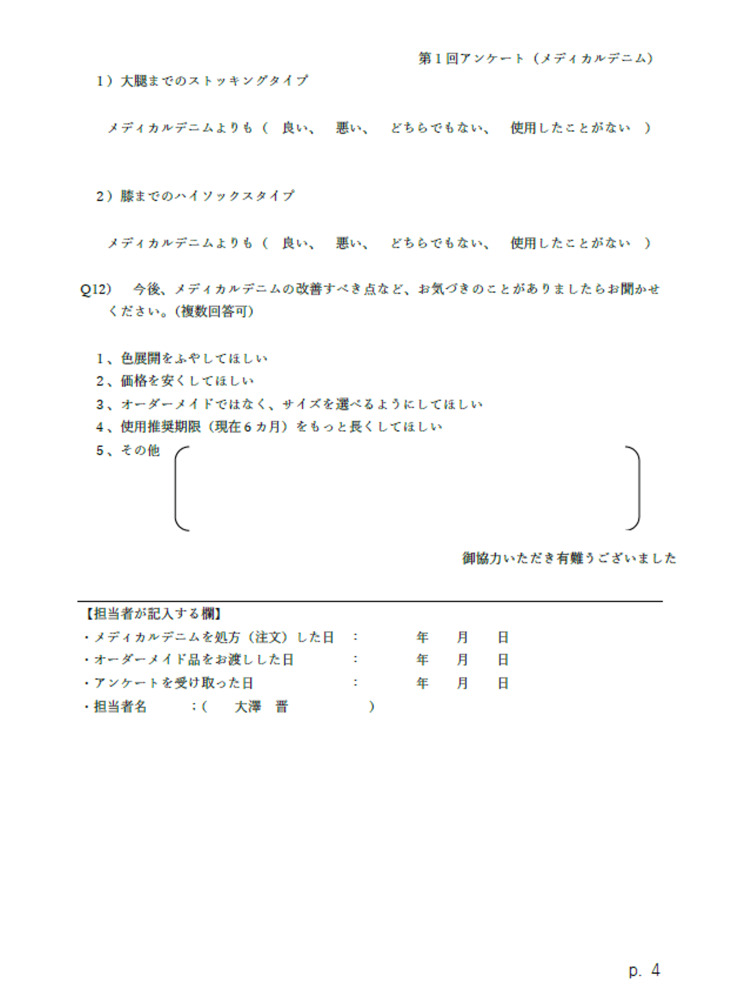
Japanese Questionnaire Page 4

**Figure 10:**
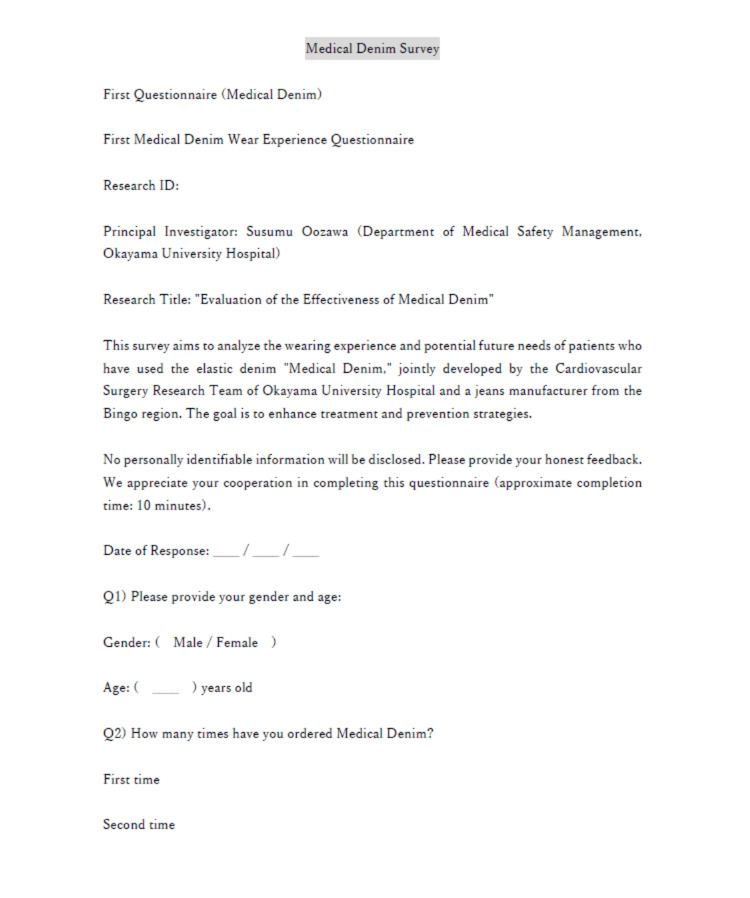
Questionnaire translated into English Page 1

**Figure 11:**
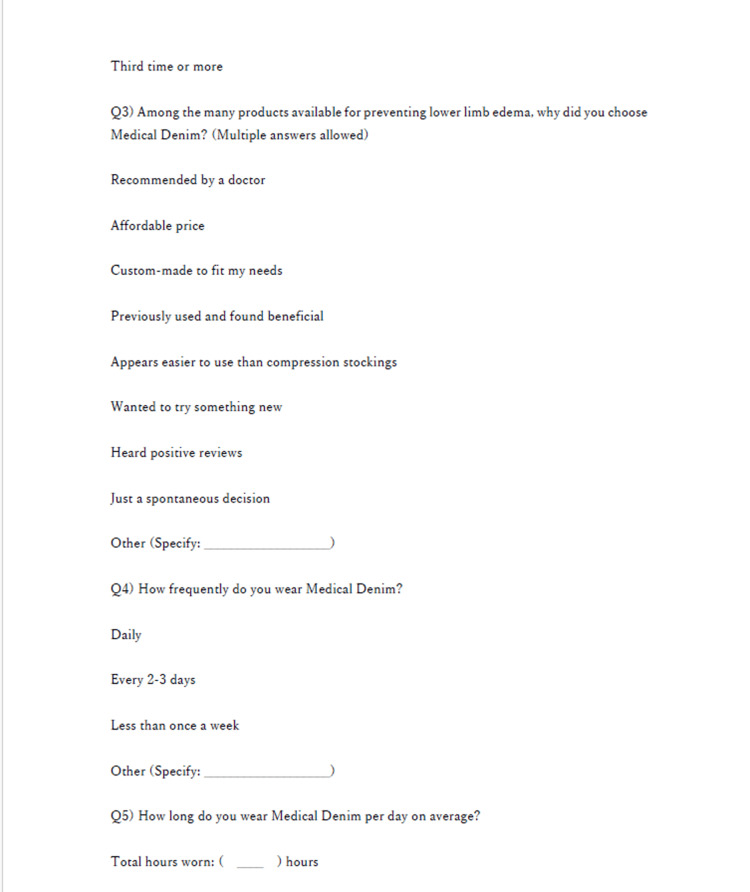
Questionnaire translated into English Page 2

**Figure 12:**
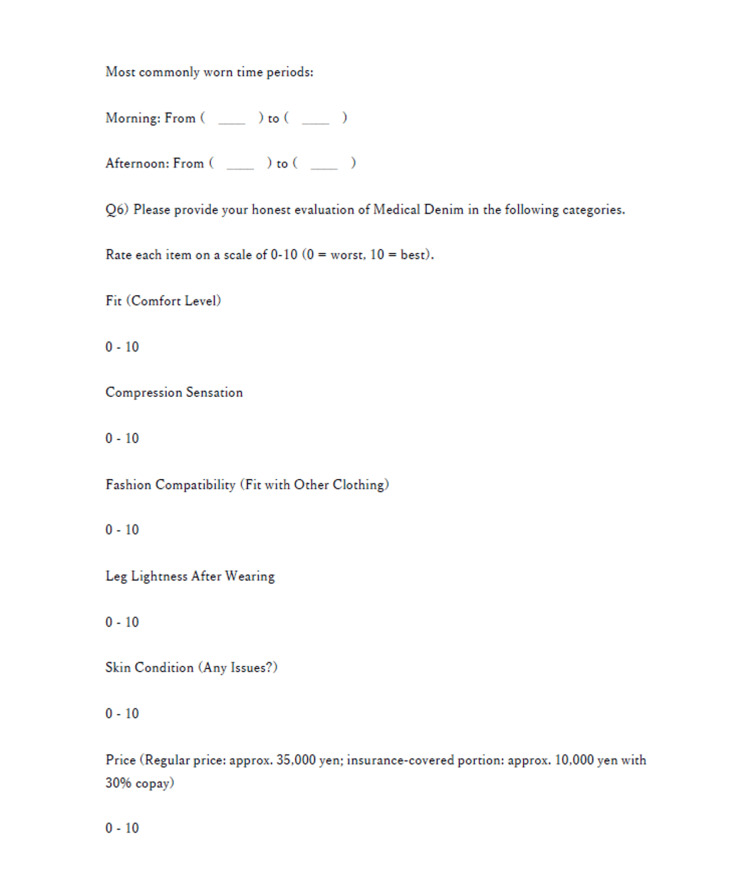
Questionnaire translated into English Page 3

**Figure 13:**
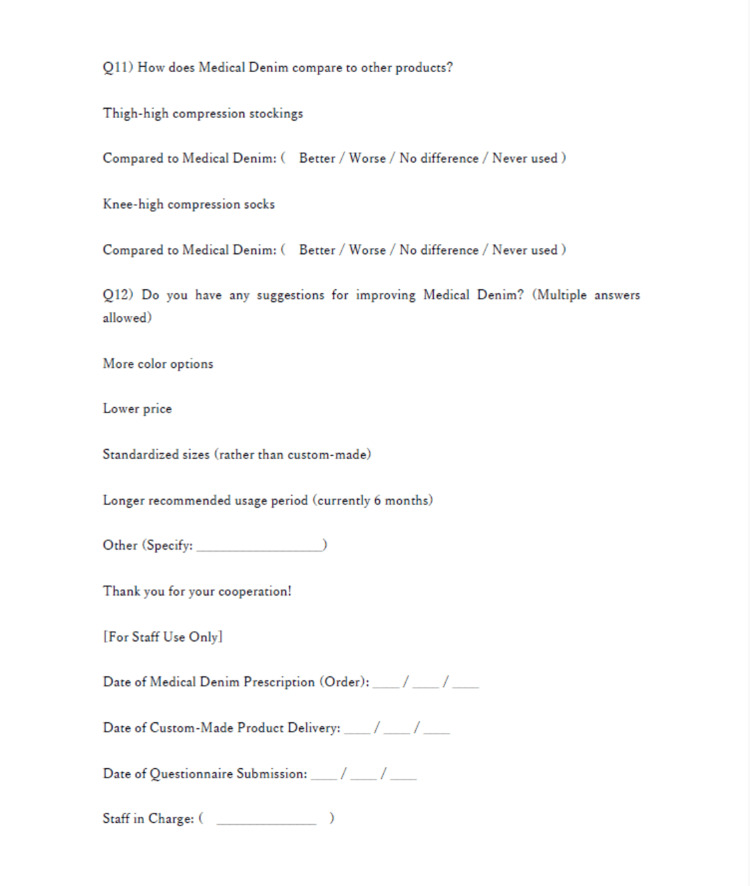
Questionnaire translated into English Page 4

## Appendices

Questionnaire in Japanese

Questionnaire translated into English
